# Ethical Concerns of the Veterinarian in Relation to Experimental Animals and In Vivo Research

**DOI:** 10.3390/ani13152476

**Published:** 2023-07-31

**Authors:** Łukasz Kiraga, Andrzej Dzikowski

**Affiliations:** 1 Division of Pharmacology and Toxicology, Department of Preclinical Sciences, Institute of Veterinary Medicine, Warsaw University of Life Sciences, 8 Ciszewski St., 02-786 Warsaw, Poland; lukasz_kiraga@sggw.edu.pl; 2 Department of Food Hygiene and Public Health Protection, Institute of Veterinary Medicine, Warsaw University of Life Sciences, 159 Nowoursynowska St., 02-776 Warsaw, Poland

**Keywords:** veterinarian, ethics, deontology, experimental animal, in vivo studies

## Abstract

**Simple Summary:**

The veterinarian’s primary responsibility is to safeguard animal health and public health, guided by the motto “Sanitas animalium pro salute homini” (the health of animals for the well-being of humans). However, there exists a distinct realm that operates separately from the core duties of veterinarians: the oversight of in vivo research. While animal studies are crucial for scientific advancement, they unfortunately involve the suffering and demise of numerous individuals. Veterinarians strive to heal animals, prevent diseases, and alleviate their pain, but in the case of animal experiments, these objectives are often unattainable. Instead of providing care, veterinarians witness the deliberate induction of various diseases, suffering, and death in animals. This situation raises ethical concerns and prompts the question of whether a veterinarian’s involvement in in vivo research conflicts with their professional deontology. This article, drawing on the definitions of ethics and deontology, examining human–animal relationships across historical periods, analyzing veterinarians’ codes of ethics in select European countries, and considering legal obligations concerning in vivo experiments, aims to provide insights and conclusions on this subject.

**Abstract:**

Animal experiments, despite their controversial nature, play an indispensable role in scientific advancement and led to numerous significant discoveries. The supervision of veterinarians in the realm of in vivo research holds immense importance. However, this particular aspect of veterinary medicine, distinct from their other activities, can pose ethical challenges. Veterinarians are entrusted with the prevention of diseases, healing, and pain elimination, yet in the case of animal experiments, they witness intentional suffering and death. This article evaluates the ethical and professional deontological aspects of this issue. It explores the historical evolution of human–animal (including experimental) relationships and discusses how deontology stems from the definition of ethics. The article also examines codes of ethics for veterinarians, providing illustrative examples. It highlights that the actions of veterinarians in this domain align with their deontology and emphasises the role of veterinarians in in vivo research as viewed within current legal frameworks. In conclusion, the veterinarian’s participation in animal research is both ethically and deontologically justified, and it is also a legal requirement.

## 1. Introduction

In vivo animal testing is undoubtely an extremely important aspect of life sciences. It is difficult to imagine the progress of biological sciences without this domain. Mankind owes many important discoveries to animal research, not the least of which we benefit from on a daily basis. For instance, smallpox was eradicated thanks to a vaccine created in research using animals [1]. One of the first in vivo studies involved penicillin. Although Ian Flemming discovered penicillin in vitro, he, along with Florey and Chain, won the Nobel Prize for research conducted on mice showing how penicillin could be used to fight infections [2]. In vivo research helped create modern vaccines against serious, often fatal, or incurable diseases, such as against polio, tuberculosis, meningococcus, and human papilloma virus, which causes cervical cancer [3]. In general, survival rates in cancer are increasing due to the in vivo studies conducted mainly in mice. An example is the discovery of herceptin, a humanised human protein that helped increase the survival rate of breast cancer patients [4]. Another example is the animal studies conducted on Tamoxifen, which resulted in a 30% decrease in mortality in breast cancer patients in the 1990s [5]. Today, many diseases, against which medicine was once helpless, are being successfully treated. An example is AIDS; through the use of highly effective antiretroviral therapy (HAART) developed with the use of animals, it is possible to almost completely eliminate the symptoms of the disease, although the carrier of the virus remains [6]. Social unawareness that therapies for common disease entities, such as asthma and type one diabetes, are possible through animal research, can be observed.

It is worth noting that the development of novel diagnostic methods and new surgical techniques is dependent on animals. Important discoveries in transplantation, regenerative medicine, or immunology are also derived from animals. Every drug, vaccine, or diagnostic substance has to go through a phase of preclinical testing before it enters the phases of clinical trials and is registered. It is safe to say that, to a greater or lesser extent, almost everyone benefits from animal research, taking advantage of the scientific achievements we owe to them. 

In vivo testing, despite the tremendous benefits it gives, is considered controversial, as evidenced by protest actions conducted in various regions of the world, or the activity of ”animal rights” organisations aimed at limiting, or even stopping, in vivo research. 

The problem of using animals in experiments raises considerable emotions. It is subject to legal regulations, including administrative rules and norms of professional ethics, scientific discourse, and a lively social debate. It is argued that the suffering of the animals involved makes animal research unethical. Therefore, it should not take place.

In recent decades, there was a growing interest among animal rights organisations and the broader community in the moral and ethical issues surrounding the use of animals in scientific research. Many express their opposition to such practices, arguing that the use of animals in experiments, especially primates, is ethically wrong and violates animal rights [7].

The main ethical argument put forward by animal rights organisations is the belief that animals have a right to dignity and protection from suffering. Scientists often subject animals to stressful conditions during experiments, which can cause pain and suffering. Some claim that there are other research methods that are just as effective and do not require the use of animals. According to their claims, in vitro tests, computer models, and experiments on human tissues are some of the many alternatives that provide results comparable to those obtained in animal studies [8].

Some also raise the issue of scientific efficacy. The results of animal studies do not always directly translate to humans, raising questions about the reliability and applicability of such experiments in biomedical research [9]. False or incomplete conclusions can not only lead to delays in scientific progress and generate economic costs, but also potentially affect human health [10]. 

Opponents of animal research also highlight the growing public disapproval of the practice. As society evolves, more and more people view the use of animals in experiments as morally unacceptable. Therefore, animal rights organisations are calling on scientists and research institutions to take a more ethical approach to conducting research [11].

However, it should be noted that the issue of animal research is not simple. There are scientists and experts who believe that in some cases animal research is necessary, provided that strict ethical standards are met and there are no alternative research methods.

This study discusses ethical issues in animal research. Research is focused on the ethical problems of those whose participation is a common element and a prerequisite for any in vivo experiment: the veterinarian. In particular, the authors intend to indicate ethical and legal factors facilitating the making of morally charged decisions by veterinarians in the case of laboratory animals [12]. 

This work constitutes a new scientific approach to the researched issue, as it tries to move away from the emotions that usually accompany the issue of animal testing. Moreover, the analysis is directed to veterinary surgeons, with the possibility of practical use of the research results. It would also be desirable to use the current study in veterinary education. Therefore, this article contains certain propaedeutic elements.

It is argued that the suffering of the animals involved makes animal research unethical. Therefore, it should not take place. This study will discuss ethical issues in animal research, focusing on the ethical problems of those whose participation is a common element and a prerequisite for any in vivo experiment: the veterinarian. In particular, the authors intend to indicate ethical and legal factors facilitating the making of morally charged decisions by veterinarians in the case of laboratory animals [12].

The authors decided to limit the area of the problem analysis to the European Union, as a representative example of the Western legal tradition and the Western cultural circle.

In the Western cultural circle, uniform concepts of science and ethics in science are observed, an element of which is the work ethics of veterinarians in animal testing. In the opinion of the authors, the results obtained, conclusions drawn, and observations made are of a general nature and can be applied on a global scale.

## 2. Ethics

In order to reliably assess the ethical aspect in the in vivo research, it is necessary to define the concept of ethics. Ethics, or moral philosophy, is a branch of philosophy concerned with moral duty: moral right vs. moral wrong. Ethics determines its detailed content: the rightness, and seeks ultimate explanations of norms, the genesis of evil, as well as the way to overcome it. In other words, ethics can be defined as an individual’s personal beliefs regarding what is right and wrong or good or bad [13].

The concept of deontology is closely related to the notion of ethics. It is one of its branches. The word deontology comes from the Greek word for duty (deon) and the word for science (logos). In the modern moral philosophy, deontology is a type of normative theory regarding which choices are morally required, prohibited, or permitted [14]. The above definitions can be merged and paraphrased: deontology is the science of the ethical means to achieve the goal. The issue of deontology particularly often refers to the proper implementation of activities related to the profession—the professional ethics. According to deontology, including veterinary professional deontology, the end should not justify the means since the ethical, moral aspect of reaching the designated goal is more important, which distinguishes this concept from consequentialism. 

Deontological rules in professional ethics are formulated with special attention to the moral aspect, but have legal norms as their basis. This also applies to veterinarians, who base the rules of deontology in their professional work on the veterinary codes of professional ethics. 

Thus, the code of ethics enables the veterinarian to choose the right, moral, and good means to achieve the goal set [15]. Even though it is difficult to define the concepts of good and evil, since they depend on the adopted value system, the code of ethics determines the fulfilment of professional activities in a good, and therefore, *ethical* way. Nevertheless, it is impossible to say unequivocally which actions are unambiguously good and which are evil, and the authors postpone such a categorical statement.

Each state introduced its own code of ethics for veterinarians, hence, professional deontology can vary depending on which country one practices the profession in. Some codes of ethics of veterinarians describe the issue of animal testing. In spite of local minor differences in veterinary professional ethics, the work ethics of veterinarians in animal testing was found to be uniform in the Western culture. Nevertheless, those codes express common general principles, and can be regarded as the unified voice of the veterinarian community on a global scale.

Undoubtedly, all of them were formed based on the view of the currently prevailing human–animal relationship [14,15,16,17,18,19].

Therefore, it is important to demonstrate how the human–animal relationship changed throughout history in order to understand how current ethical standards for veterinarians practicing in laboratory animal medicine formed.

### 2.1. Human-Animal Relations throughout History as Viewed by Various Philosophical Schools

The earliest historical rationale for the relation–animal view is at least as early as Pythagoras in 4th–5th century BC [20,21,22]. His belief in the metempsychosis, inspired by Orphism, resulted in the promotion of vegetarianism. It is believed that he claimed that as long as men would kill animals, they would kill each other. This view can be still found today in several Far Eastern cultures and religions. 

Aristotle indicated that a hierarchy of entities exists: inanimate things, plants, animals, and humans at the top. Thus, according to Aristotle, animals have a lower ontic status than humans, and help mankind achieve their own goals. He claimed that animals do not behave morally and rationally, but are capable of feeling. He justified killing animals only when it served to achieve higher goals [20,22,23,24].

Aristotle inspired Thomas Aquinas, who argued that less-perfect entities should serve those, who are more perfect, referring to the human–animal relationship. This view still prevails in Christian countries, and thus, Western legal tradition today. On the other hand, Francis of Assisi promoted the view that animals are perfect as an act of His creation. He encouraged the affirmation of nature; this view, however, was marginal, and did not leave much of a mark on the human–animal relationship [7,22,25]. 

Descartes promoted the concept of “machine” [22,26]. According to him, animals do not have vitam sensumque, which makes them incapable of feeling, including feeling pain. Animals do not suffer, while their reactions to pain are simply reflexes to damaging agents. Nevertheless, this period of such an approach to animals led to a flourishing of the animal research, most of which was conducted inhumanely and caused great suffering (e.g., vivisection). 

It is known today how wrong the Cartesian theory of the time was. The Cambridge Declaration on Consciousness, for example, signed by a group of leading scientists attending the Francis Crick Memorial Conference at the University of Cambridge in the UK in July 2012, recognises that animals, including mammals, birds, and some other vertebrates, are conscious beings capable of experiencing emotions, cognitive abilities, and awareness. The declaration highlights the neuroanatomical similarities between the brains of these animals and humans, supporting the idea of consciousness. It emphasises the ethical importance of considering the welfare and treatment of animals in scientific research and human activities [27]. Moreover, there are scientists who associate still other attributes with animals. Prominent primatologist and ethologist, Frans de Waal proved through his numerous behavioural studies that mammals even demonstrate dissociate emotional and cognitive empathy [28], whereas according to famous biologist and behavioural ecologist Marc Bekoff, animals other than humans are able to exhibit moral and emotional intelligence [29].

In opposition to Descartes’ claims stood John Locke. According to him, cruelty to animals negatively affects human morality. He promoted a view that animals can be used for human purposes, provided that respect is given and proper treatment guaranted [20].

Rousseau argued that all organisms (humans and animals alike) fight for their survival. In his view, animals do not behave rationally, but they are undoubtedly conscious, and similar to humans, are subject to the laws of nature. He emphasised the superiority of men over other creatures, but commanded to give them respect. 

Kant created the concept of the categorical imperative, which was intended to be a set of universal ethical norms and laws applicable to every human being. Human actions should serve the general public and have no selfish motives, but only in relation to thinking beings, which, according to Kant, include only humans. He argued that man is an end in itself, and the animal is the way to an end [20,30].

Schopenhauer was of the opinion that animals have the ability to feel and behave rationally, but to a narrower degree than humans, which was close to current views [20]. 

The present human–animal relationship, including in the context of the in vivo research, is most likely due to Bentham’s concept of utilitarianism, according to which, every action taken should be evaluated in terms of its consequences. What is useful is good. It does not mean, however, that unethical behaviour in relation to animals is acceptable [20,22,31].

According to Bentham, the determinant is not thinking, but feeling, and animals are undoubtedly capable of feeling. Thus, they can be used, provided that it serves a good purpose, and keeping in mind their ability to feel and suffer. This view shaped the modern human–animal relationship (concerning also animal testing), and still prevails today [20,24,32,33,34]. 

Contemporary philosophical, sociological, and anthropological concepts can also be mentioned [30,35,36]. Nevertheless, the authors do not intent to provide a complete anthropological or sociological theory, nor a philosophical system. Current analysis is not an anthropology of science, nor is it a sociology of science, of the in vivo research, or of the veterinary profession.

The discussed views influenced the content of the codes of professional ethics of veterinary surgeons, which directly determine the deontology of veterinarians. The actions of veterinarians, including those related to the in vivo research, must firstly comply with the applicable law, and secondly, must be ethical. Legal norms are derived from current legislation, while ethical norms are acquired from the code of ethics. Thus, the concept of ethics depends on the norms contained in the code, and its axiological basis. It is a system of connected vessels.

The following chapter will discuss ethical standards for veterinarians who deal with animal testing, according to the representative examples of the veterinary codes of ethics enforced by the European Union.

### 2.2. Ethical Standards for Animal Testing According to Codes of Ethics in Force in Various States

As was revealed, the performance of experiments by veterinarians is influenced by philosophical trends shaping the relationship between man and laboratory animals [31,37], as well as various types of legislation. Nevertheless, the veterinarian remains a physiscian, and a member of the profession of public trust. Therefore, he/she is obliged to observe not only their own particular system of ethics, resulting from their own philosophical or religious beliefs. Every veterinarian must comply with professional ethics and deontology, as defined in codes of professional ethics. Failure to comply with it is subject to professional liability. One may lose his/her license to practice veterinary medicine in the case of breaches of the law regarding animal experiments, the rules of the medical art, or moral standards.

In various countries and states, veterinary professional corporations and veterinary social organisations, or other representatives of the veterinary profession [14,38,39,40] have different approaches to the regulation of the discussed issue. Conducted analysis, however, revealed that all these differences are of secondary importance. General principles of practicing the veterinary profession, and of conducting the in vivo animal research, were found to be universal in the Western society.

Selected representative regulations of states of the European Union, and thus, Western cultural circle, were examined. These are states at a comparable level of civilisation, common ethical, and legal sources (common history, religion, and philosophy). These states are characterised by recognition of the same rules for conducting scientific research and scientific discourse [41,42].

#### 2.2.1. Types of Regulation

A comparative ethical and legal analysis was carried out. It should be noted that the standards of professional ethics included in the form of codes are legal standards, for non-observance, of which there is a risk of liability.

The conducted research allowed us to indicate that there are three possible forms of regulation of the professional ethics of veterinarians in the scope of laboratory animal veterinary medicine:

- Tacit form;
- Mention;
- Special regulation.

In the type designated as “tacit regulation”, the issues of professional ethics in animal research are not regulated separately in the code of ethics. They are rather subject to general principles, such as lege artis performance, accordance with the law, and scientific knowledge. An example of such a type is France [43,44,45,46].

In the type defined as “mention”, the issues of professional ethics in animal research are regulated in the code of ethics only to a meager extent. Typical is one short provision of this scope of professional activity. An example is the German code of veterinary professional ethics [20,40,47,48], according to which veterinary surgeon must support scientific progress in all fields of medicine and life sciences. Every veterinarian ought to support the research, development, and use of alternatives to animal experimentation, an care for animal welfare.

In the type described as the “special regulation”, the issues of professional ethics in animal research are regulated separately in the code of ethics, which devotes a lot of attention to them. 

In the further part of the current study selected, representative regulations in this regard will be analysed.

#### 2.2.2. Example of the Specific Regulation: Poland

The Polish code of veterinary ethics and deontology devotes Chapter IV, Articles 31–33, to the issue of laboratory activity [49]. The premises, which allow for the participation of a veterinarian in scientific animal research and in the implementation of scientific achievements, are specified. Veterinarians’ activity in animal tests is acceptable only if there are reasonable grounds that it will not be harmful to humans, animals, and the environment; if experimentation using live animals is necessary for diagnostics, teaching, or for scientific research, that is expected to be of benefit to human or animal health; and if the applicable legal provisions are observed [50], there are cumulative conditions. It should be, therefore, interpreted that any activity that does not meet all of these conditions is unlawful, illegal, and most of all, unethical.

In addition, the veterinarian is burdened with an ethical obligation to watch over the entire course of the experment. He/she has, therefore, a moral and professional responsibility for all researchers [14] and for everything that happens during the experiment. The veterinarian participating in experiments on animals, or supervising their course, is obliged to ensure that unnecessary pain, suffering, and injuries are not caused to animals [32].

#### 2.2.3. Example of the Specific Regulation: Italy

Article 39 of the Italian code of veterinary ethics is devoted to the discussed problem [51]. Veterinarians should strive for the progress of medicine and science. It is recognised as the highest goal. Other goals, such as the welfare of mankind or animal welfare, are of intermediate importance.

To ensure this, the Italian laboratory veterinarian must rely on four criteria. These are the top criteria for ensuring the quality of research and their moral justification. They are important both from the point of view of the society, veterinary profession, science, and the veterinarian him/herself.

The first criterion refers to the earlier scientific research, improvement of knowledge, and activities taken up to protect the health and welfare of animals and humans. The second criterion is the obligation to comply with legal norms. The third is the welfare of laboratory animals (the 3R principle, the development of alternative methods, and appropriate means to avoid unnecessary suffering) [52]. Finally, the fourth criterion is the possibility of using the conscientious objection. 

#### 2.2.4. Example of the Specific Regulation: Spain

Professional deontology of Spanish veterinarians in relation to the use of laboratory animals is described in Chapter XII, Article 29, of the local code of ethics [53,54].

Spanish veterinarians cannot conduct experiments on live animals without an authorising consent of the competent state authority. All animal experiments must be based on generally recognised scientific standards. Among these scientific principles, the 3R principle is directly mentioned. Minimizing the number of animals used in the experiment is also emphasised. Further regulations (Article 29, sections 5–6) concern not as much laboratory animals as experiments in the clinical phase. 

#### 2.2.5. Other Factors

The detailed examples analysed, discussed, and presented above allow us to draw conclusions of a general nature. Although these are local regulations from the area of given European states, they can be considered as an exemplification of general global rules of professional ethics that apply to all veterinarians around the world, regardless of jurisdiction.

It should be noted that in most states, the ethical standards applicable to veterinarians can be derived indirectly from the provisions of the acts on animal experimentation. There are also international regulations, such as those of the European Union, the analysis, and legal interpretation, of which will be presented in the further part of the current paper. 

It should also be noted that in addition to the above sources of legal standards, a given laboratory veterinarian can be also obliged to observe scientific ethics as an employee of laboratories operating within research institutes or universities [15]. 

In connection with all of the above, a gap, or conflict, between the need for evidence-based medicine and ethics may occur [55]. This may give rise to serious ideological dilemmas and may practically affect the quality of the work in the laboratory or animal facility. 

Moreover, a discrepancy between the morality (philosophical, religious, etc.) of a given person, a given veterinarian, or a given society and veterinary professional ethics may be observed. Discrepancy between what the law allows and what ethics indicates may be revealed. 

A significant problem is the “detachment” or “abruption” from laboratory animals by the veterinary surgeon. It is difficult for him/her, and psychologically destructive, but at the same time, it serves the objectivity of research. Each veterinarian, therefore, must balance his/hers responsibilities rising from different value systems, and choose the solution that is most compliant with the law and with the ethical standards. The instrument of this choice may be the scientific knowledge acquired in the course of studies, specialisation, or courses devoted to laboratory issues.

## 3. European Law Regulating In Vivo Animal Testing

Professional deontology, including that of a veterinarian, is not only conditioned by ethics prevailing in a given society, but also by its religion, history, and law. The veterinary code of professional ethics cannot be in conflict with the law in force. Standards of professional ethics for veterinarians, as well as legal regulations, must be consistent in democratic countries. This is the standard of the Western cultural circle. 

In addition to the regulations of individual states, more general legal provisions with an international scope exist. They firlmy affect state laws. In this paper, the authors decided not to examine the laws of individual states. Instead, the European Union regulations will be analysed as a meaningful, and as a representative example of universal significance.

Currently, the overriding legal act in the European Union member states is the Directive 2010/63/EU of the European Parliament and of the Council of 22 September 2010 on the protection of animals used for scientific purposes [56,57,58].

As a result of its entry into force, member states had to introduce national regulations implementing all aspects of the directive within five years. Thus, legal regulations in individual member states regarding the issue of animal testing are conditioned by various legal acts, the common element of which is the realisation of the same crucial elements arising from the directive [40].

Therefore, the provisions of the legal acts in force in individual member states are uniform because their assumptions and axiological foundations are convergent. There is no point in analysing separately the legal acts of each member state, since their assumptions coincide. 

Instead of the analysis of particularities, the authors will focus on the legal role of the veterinarian in scientific research according to Directive 2010/63. This legislative act consists of 56 points that make up the preamble, 66 articles grouped into 6 chapters, and 8 annexes. 

Article 25 stipulates that member states shall ensure that each breeder, supplier, and user has a designated veterinary surgeon with expertise in laboratory animal medicine, or, if it would be more appropriate, an ”expert” with appropriate qualifications performing advisory duties on animal welfare and treatment. 

Thus, establishment, regardless of its form of operation, must employ a veterinarian with relevant expertise in laboratory animal medicine or an ”expert with relevant qualifications”. It is not clear what specific qualifications, skills, knowledge, or education this ”expert” should have. Nevertheless, this position is treated as an alternative to a veterinarian to perform veterinary supervision at the establishment.

The authors hereby postulate that the ambiguous position of the ”expert” should be removed from the normative acts. Moreover, in states where there is an official title of a veterinary surgeon (specialist in laboratory animal medicine), this function ought to be limited to only veterinarians who can demonstrate such a national or European specialisation.

Another reference to the role of the veterinarian in the in vivo research can be indicated in Article 9 on obtaining wild animals for research. The norm states that wild animals may be obtained for research only under the condition of scientific justification, confirming that the purpose of the procedure cannot be obtained using an animal bred for use in procedures [59].

In addition, the acquisition of wild animals shall be carried out by competent persons using methods that do not cause the animals avoidable pain, suffering, distress, or permanent damage. All animals found to be injured or in poor health during or after the capture shall be examined by a veterinarian. They should then be subject to appropriate procedures to minimise suffering. Thus, the presence of a veterinarian is indispensable when acquiring wild animals for research, because if an animal is injured, it must be properly examined and treated [60]. This requirement can be derived not only from the statutory regulations, but also from the scientific veterinary knowledge, and the veterinary professional ethics.

Another role of the veterinarian is mentioned in Article 16, concerning the use of animals in an experiment once they are subjected to procedures. This is only possible if four conditions are met:

- If the severity of the previous procedures was mild or, at most, moderate (the severity classification of the procedures is referred to in Annex VIII);
- If it is shown that the animals fully recovered their general health and welfare;
- If the subsequent procedure is classified as mild, moderate, or terminal without recovery, and;
- It is in accordance with veterinary advice, taking into account the animals’ life experience.

It is possible to deviate from the first condition; after the examination performed by a veterinarian, the animal can also be reused if it was subjected to a severe procedure, but not more than once. Thus, it is for the veterinarian to make the final decision whether the animal can be reused in procedures. Such a decision cannot be made without his participation. In the authors’ opinion, this is a reasonable normative solution. Only a veterinary surgeon has sufficient competence, knowledge, and skills to adjudicate on the health of the animal. Not only is the knowledge of laymen (outside observer) not enough, they also perceive science differently than researchers and veterinary professionals [36].

Article 17 considers the role of the veterinarian. It regulates the fate of animals after the procedures performed on them are completed. According to paragraph 2, the veterinarian, or another competent person, makes the decision to either kill, or keep the animal alive. It is, however, not clear what competence the “other competent person” must have to perform a function equivalent to the veterinarian in this case. Such a regulation is assessed by the authors definitely negatively. This is a potential gateway to abuse.

An animal is killed if there is a probability that it will continue to feel moderate or severe pain, suffering, or distress, or that it will experience permanent damage. This means that the veterinarian, having examined the health of the animal after the procedures, decides whether the animal can be left alive or should be killed due to the suffering it is experiencing.

Article 26 describes the connection between the veterinarian and the functioning of the animal welfare advisory body. According to the statutory provisions, each establishment must set up a welfare advisory body, which must include (at least) a person or persons responsible for the animal welfare and animal care, and in the case of a user, an academician. 

The animal welfare advisory body receives information from the designated veterinarian, or expert referred to in Article 25. Thus, it can be stated that the veterinarian (or the aforementioned “expert”) cooperates with the welfare body without being a member of it by him/herself. His/her role is providing information on animal welfare, emphasizing the assessment of animal health.

Each establishment must employ a “designated veterinarian” with relevant expertise in laboratory animal medicine, where he or she provides extensive veterinary services. 

In light of the discussed directive, the role of the veterinarian in the in vivo research concerns: deciding whether to reuse animals in procedures, determining whether to keep animals alive or kill them after procedures are completed, and cooperating with the welfare body in implementing animal welfare. These activities, for which there is a legal requirement that they are carried out specifically by a veterinarian, are hereby considered as “direct” participation of a veterinarian in the in vivo research. 

An “indirect” participation can also be distinguished: when specific functions are performed by a veterinarian, even though legally they do not have to be performed by him/her. Examples include the so-called animal facility famanager and personnel of the establishment.

As for personnel of the establishment, Article 23 of the discussed European Directive distinguishes between those who perform the functions of:

- Performing procedures on animals;
- Planning procedures and projects;
- Taking care of animals; and
- Killing animals.

Persons performing these functions must be trained in the scientific discipline relevant to the work being undertaken, and should have knowledge of the species in question. The scope of mandatory training for these individuals is detailed in the Appendix V. 

The animal facility manager is an informal term, not appearing in the normative text. The animal facility manager performs the functions set forth in Article 24.1, and thus is responsible for supervising the welfare and care of the animals; as well as ensures that the personnel handling the animals have access to information on the specific species housed in the facility. Moreover, he/she is responsible for ensuring that the personnel is properly educated, competent, and continuously trained, as well as supervised till the required skills are demonstrated. The establishment may have one or more persons performing this function.

Although there is no legal requirement that any of the staff or manager positions be held by a veterinarian, they are often held precisely by veterinarians because of their knowledge experience in working with animals.

There is another legal aspect tying the veterinarian to in vivo experimentation. This is alluded to in Article 34, which deals with the obligation of member states to inspect breeders, suppliers, and users. 

This article provides rules for determining the frequency of such inspections. In most of the European Union member states, the veterinary inspection is involved, and the persons directly inspecting the centers are veterinary officers (and thus, veterinarians). They inspect the legality of the conduct of in vivo experiments; that is, whether they are carried out in accordance with the law currently in force (resulting from the directive), and prepare the relevant inspection documentation, which must be kept for at least five years.

The significant role of ethical committees in the process of conducting animal tests should be indicated. Such committees decide on admission to experiments, on the number of animals, or on the end of the procedure. It was found that often, but not always, a veterinary surgeon is a member of such a committee. As already indicated, according to general legal principles (including logic and axiology of the veterinary law), veterinarians are the only people who have the right to make judgments about, and to assess the health of animals. The authors hereby propose that in each ethical committee, at least one veterinary professional should be included; preferably, veterinarians experienced in animal testing and specialised in laboratory animals’ health and pathology [61].

The legal provisions, however, only seem to constitute directives of conduct. The regulations themselves do not indicate to the veterinarian what ethical decisions or what choices to make [12]. Nevertheless, if, when interpreting a legal provision, he/she takes into account the ethical background and axiological grounds on which these norms are based, then making the right decision will be easier.

Furthermore, in the opinion of the authors, ethical obligations should not be created in abstracto, or metaphorically “in vitro”, in isolation from the real users of ethics (veterinarians), from their limitations, or the possibility of meeting the requirements. In the authors’ opinion, professional ethics and veterinary law are not ideal or idealistic theoretical concepts, but tools for practical application. Therefore, law and professional deontology must take into account the practical eventualities and limitations of the users.

## 4. Conclusions

In vivo animal research undeniably has a huge impact on the development of science, and these scientific achievements are of great value to humans. However, it is important to note that not all experiments involve equal animal suffering. We have four categories of the severity of procedures: non-recovery, mild, moderate, and severe [56]. At the planning stage of an experiment, we have an obligation to implement the 3Rs principle, one component of which is ”refinement”, according to which we must strive for the least possible animal suffering. However, there are some experiments in which, due to the type of research, we cannot avoid causing severe pain to animals, and this raises more ethical questions: does the desire to gain new knowledge and discoveries morally justify harming animals? This is still a controversial point. According to the principle of refinement, in in vivo research, we should also strive to use animals from the lower rungs of the evolutionary ladder. Nevertheless, many experiments are being conducted on primates, which are evolutionarily closest to humans. There are still conditions with unknown pathogenesis and effective treatment, such as Alzheimer’s or Parkinson’s disease, and mouse or rat models of these diseases do not advance our knowledge; in order to finally discover effective methods of preventing these diseases, should we conduct research on primates, which is the most sentient group of animals? This is an ethical dilemma.

The role of the veterinarian in in vivo animal research is crucial. As demonstrated, a veterinarian is the only person who has enough knowledge, skills, and legal authorisation to assess the health of laboratory animals. 

Moreover, a veterinary surgeon must be involved in the scientific research because, as the legal norms state, he/she must be employed at each animal facility, must decide whether animals are to be left alive after procedures, must have a welfare advisory body cooperating with him/her, must approve the reuse of animals in procedures, and must oversee the acquisition of wild animals for research. 

It is not precluded for a veterinarian to participate in the scientific research as a so-called animal facility manager (Directive 2010/63, Article 24.1), or a staff member directly involved in the conduct of an experiment. Performing any of these functions is not unethical, as it does not contradict a veterinary code of ethics. Most of such codes explicitly state that veterinarians should strive for the proper conduct in any field (including animal experiments), and ensure humane treatment of all animals (including experimental animals). Killing animals at the end of an experiment (which is at the discretion of the veterinarian) may raise moral questions, but leaving an animal suffering alive would be far less morally correct than euthanasia, and is therefore ethically correct. 

The participation of veterinarians in the discussed kind of research is legal, necessary, and should not pose major ethical problems, as it does not contradict the codes of ethics of a veterinarian. Direct participation in the in vivo experiment should also not raise questions about the ethicality of such conduct, since this issue is overseen by Ethics Commissions operating in each member country [61].

Such commissions authorise only experiments in which the suffering of animals is as low as possible and morally justified; tests that may entail the acquisition of knowledge useful to humans. These provisions, therefore, justify the use of animals in scientific research according to utilitarianism. 

Ethics committees do not approve badly planned or unethical experiments. Thus, taking part in a legitimate experiment, one is assured that it does not contradict ethics, nor deontology. Whether or not it is morally correct depends on the value system one adopted, and how it will balance the different responsibilities that come from different normative sources. The ethical and axiological background of the law, including the law of professional ethics, as well as scientific veterinary knowledge in the field of health and welfare of laboratory animals can be an aid and decisive instrument.

## Data Availability

Data sharing not applicable.

## References

[1] Riedel S. (2005). Edward Jenner and the History of Smallpox and Vaccination. Bayl. Univ. Med. Cent. Proc..

[2] Gaynes R. (2017). The Discovery of Penicillin—New Insights After More Than 75 Years of Clinical Use. Emerg. Infect. Dis..

[3] Koff W.C., Rappuoli R., Plotkin S.A. (2023). Historical Advances in Structural and Molecular Biology and How They Impacted Vaccine Development. J. Mol. Biol..

[4] Slamon D. (2000). Herceptin**^®^**: Increasing survival in metastatic breast cancer. Eur. J. Oncol. Nurs..

[5] Jordan V.C. (2014). Proven value of translational research with appropriate animal models to advance breast cancer treatment and save lives: The tamoxifen tale. Br. J. Clin. Pharmacol..

[6] Gazzard B.G. (2009). A Decade of HAART: The Development and Global Impact of Highly Active Antiretroviral Therapy. HIV Med..

[7] Gluck J.P. (2016). Voracious Science and Vulnerable Animals: A Primate Scientist’s Ethical Journey; Animal Lives.

[8] Greek J.S., Greek C.R. (2004). Replacing the Animal Model in Biomedical Research and Education. What Will We Do If We Don’t Experiment on Animals? Medical Research for the Twenty-First Century.

[9] Knight A., Bailey J., Balcombe J. (2006). Animal Carcinogenicity Studies: 2. Obstacles to Extrapolation of Data to Humans. Altern. Lab. Anim..

[10] Knight A. (2007). Systematic Reviews of Animal Experiments Demonstrate Poor Human Clinical and Toxicological Utility. Altern. Lab. Anim..

[11] 52% of American Public Opposes the Use of Animals in Scientific Research. https://speakingofresearch.com/2018/08/30/52-of-american-public-opposes-the-use-of-animals-in-scientific-research/.

[12] Vettical B.S. (2018). An Overview on Ethics and Ethical Decision-Making Process in Veterinary Practice. J. Agric. Environ. Ethics.

[13] Fischer J. (2004). Social Responsibility and Ethics: Clarifying the Concepts. J. Bus. Ethics.

[14] Herzog H. (2002). Ethical aspects of relationships between humans and research animals. ILAR J..

[15] Ashall V., Hobson-West P. (2018). 45. The Vet in the Lab: Exploring the Position of Animal Professionals in Non-Therapeutic Roles. Professionals in Food Chains.

[16] Sturma D., Sturma D. (2022). Tierversuche: Eine Ethische Perspektive. Natur, Ethik und Ästhetik.

[17] Kaleta T. (2017). Geneza dobrostanu zwierząt—Aspekty historyczne i filozoficzne. Przegląd Hod..

[18] Rollin B. (2008). Animal Ethics and the Law. Mich. Lawe Rev. First Impr..

[19] Schmidt K. (2011). Concepts of Animal Welfare in Relation to Positions in Animal Ethics. Acta Biotheor..

[20] Riether E., Weiss M.N. (2012). Tier—Mensch—Ethik.

[21] Krajewski W. (2005). Filozofia Przyrody jako Pomost Między Naukami Przyrodniczymi a Filozofią. Rocz. Filoz..

[22] Tyburski W. (2011). Człowiek—Środowisko przyrodnicze w świetle wybranych stanowisk filozoficznych i ekofilozoficznych. Paedagog. Christ..

[23] Sorabji R. (1993). Animal Minds and Human Morals: The Origins of the Western Debate.

[24] Anderson L.C., Fox J.G., Otto G.M., Pritchett-Corning K.R., Whary M.T. (2015). Laboratory Animal Medicine.

[25] Dietl A. (2021). Ortodoksja i herezja w relacjach ludzi i zwierząt. SRZ.

[26] Switankowsky I. (2000). Dualism and its importance for medicine. Theor. Med. Bioeth..

[27] The Cambridge Declaration on Consciousness. https://fcmconference.org/img/CambridgeDeclarationOnConsciousness.pdf.

[28] De Waal F.B.M., Preston S.D. (2017). Mammalian empathy: Behavioural manifestations and neural basis. Nat. Rev. Neurosci..

[29] Bekoff M. (2002). Minding Animals: Awareness, Emotions, and Heart.

[30] Hache E., Latour B. (2010). Morality or Moralism? An Exercise in Sensitization. Common Knowl..

[31] Yeates J.W. (2009). Response and responsibility: An analysis of veterinary ethical conflicts. Veter. J..

[32] Carbone L. (2004). What Animals Want: Expertise and Advocacy in Laboratory Animal Welfare Policy.

[33] Jedynak S. (2008). The human-animal relationship in its ecological aspect. Probl. Sustain. Dev..

[34] Lemańska A. (1997). Praktyczna Filozofia Przyrody Alternatywą klasycznej filozofii przyrody? Kryzys klasycznej filozofii przyrody w XX wieku. Stud. Philos. Christ..

[35] Latour B. (1983). Give Me a Laboratory and I Will Raise the World. Science Observed: Perspectives on the Social Study of Science.

[36] Latour B., Woolgar S. (1986). Laboratory Life: The Construction of Scientific Facts.

[37] Morgan C.A., McDonald M. (2007). Ethical Dilemmas in Veterinary Medicine. Veter.-Clin. North Am. Small Anim. Pract..

[38] Kaliste E. (2007). The Welfare of Laboratory Animals.

[39] Pullen S., Gray C. (2006). Ethics, Law, and the Veterinary Nurse.

[40] Binder R., Grimm H., Alzmann N. (2013). Wissenschaftliche Verantwortung im Tierversuch. Ein Handbuch für die Praxis.

[41] Micińska-Bojarek M. (2012). Europejski standard doświadczeń na zwierzętach. Aspekty humanitarno-prawne. Przegląd Prawa Ochr. Sr..

[42] Gajewska G. (2015). O w?adzy ludzi nad zwierz?tami w kulturze zachodniej—Perspektywa posthumanistyczna. Stud. Eur. Gnes..

[43] Code de Déontologie. https://www.veterinaire.fr/la-profession-veterinaire/la-reglementation-professionnelle/code-de-deontologie.

[44] Sirois M. (2022). Laboratory Animal and Exotic Pet Medicine: Principles and Procedures.

[45] Wolfensohn S., Lloyd M. (2003). Handbook of Laboratory Animal Management and Welfare.

[46] Dolan K. (2007). Laboratory Animal Law: Legal Control of the Use of Animals in Research.

[47] Ethik-Kodex Der Tierärztinnen Und Tierärzte Deutschlands. https://www.bundestieraerztekammer.de/btk/ethik/.

[48] Boscheinen J. (2020). Buchrezension zu: Kursbuch Bioethik. Biospektrum.

[49] Kodeks Etyki Lekarza Weterynarii. https://vetpol.org.pl/dmdocuments/Kodeks%20etyki%20lekarza%20weterynarii.pdf.

[50] Ach J.S. (2019). Umgang mit Tieren in der Forschung. Grundsätze des neuen Leitbilds der Universität Münster. Z. Für Evang. Ethik.

[51] Nagasawa T., Kimura Y., Masuda K., Uchiyama H., Consiglio Nazionale FNOVI Codice Deontologico. https://www.fnovi.it/fnovi/codice-deontologico.

[52] Baumgaertner H., Mullan S., Main D.C.J., Bsc H.B., Bvms S.M., BVetMed D.C.J.M. (2016). Assessment of unnecessary suffering in animals by veterinary experts. Veter. Rec..

[53] Código Deontológico Para El Ejercicio de La Profesión Veterinaria. https://colegioveterinarios.net/wp-content/uploads/2018/10/BORRADOR-C%C3%93DIGO-DEONTOL%C3%93GICO-PARA-EL-EJERCICIO-DE-LA-PROFESI%C3%93N-VETERINARIA-Versi%C3%B3n-Septiembre-de-2018.pdf.

[54] García J.M.G. (2010). Laboratory medicine and the identity change of veterinary medicine in Spain at the turn of the twentieth century. Dynamis.

[55] Vandeweerd J.-M., Kirschvink N., Clegg P., Vandenput S., Gustin P., Saegerman C. (2012). Is evidence-based medicine so evident in veterinary research and practice? History, obstacles and perspectives. Veter. J..

[56] Directive 2010/63/EU of the European Parliament and the Council of 22 September 2010 on the Protection of Animals Used for Scientific Purposes. https://eur-lex.europa.eu/legal-content/EN/TXT/HTML/?uri=CELEX:32010L0063&qid=1687795274615.

[57] Olsson I.A.S., da Silva S.P., Townend D., Sandøe P. (2016). Protecting Animals and Enabling Research in the European Union: An Overview of Development and Implementation of Directive 2010/63/EU. ILAR J..

[58] Chlebus M., Guillen J., Prins J.-B. (2016). Directive 2010/63/EU: Facilitating full and correct implementation. Lab. Anim..

[59] Skorupski J., Budniak M., Śmietana P. (2016). Etyka badań nad dzikimi zwierzętami nocnymi w kontekście oddziaływania zanieczyszczenia światłem i zanieczyszczenia hałasem. Stud. i Mater. CEPL w Rogowie.

[60] Tannenbaum J. (1999). Ethics and Pain Research in Animals. ILAR J..

[61] Verschuere B., Autissier C., Degryse A.D., Gallix P., Gottis B., Laurent J., Leinoe M., Peyclit I. (2000). Ethics committee recommendations for laboratory animals in private research in France. Lab. Anim..

